# Attributable Fractions of Risk Factors for Cardiovascular Diseases

**DOI:** 10.2188/jea.JE20100081

**Published:** 2011-03-05

**Authors:** Atsushi Hozawa

**Affiliations:** 1 Department of Public Health, Yamagata University, Graduate School of Medical Science, Yamagata, Japan; 2 Division of Epidemiology, Tohoku University, Graduate School of Medicine, Sendai, Japan

**Keywords:** cohort study, cardiovascular diseases, population attributable fraction

## Abstract

**Background:**

Cardiovascular disease (CVD) is a leading cause of death in Japan. To reduce the threat of CVD, it is important to identify its major risk factors. The population attributable fraction (PAF) is calculated from the prevalence and relative risk of risk factors and can be used to estimate the burden of these factors with respect to CVD. We analyzed the findings from several prospective studies to determine the PAFs of CVD.

**Methods:**

PAF was calculated as pd × (multiadjusted relative risk − 1)/multiadjusted relative risk, where pd is the proportion of patients exposed to that risk factor category, according to data from the Ohsaki Cohort Study, EPOCH-JAPAN, NIPPON DATA80, Miyagi Cohort Study, CARDIA Study, and ARIC Study.

**Results:**

Nonoptimal blood pressure explained 47% and 26% of CVD mortality in middle-aged and elderly Japanese, respectively. Cigarette smoking explained 34% of all-cause mortality in middle-aged men. The combination of hypertension and cigarette smoking explained 57% and 44% of CVD mortality in younger men and women, respectively. Furthermore, the presence of at least 1 nonoptimal risk factor explained most CVD deaths and all-cause deaths.

**Conclusions:**

Established CVD risk factors, especially high blood pressure and cigarette smoking, explained a large proportion of CVD mortality and all-cause mortality. Prevention, early detection, and treatment of these conventional risk factors are required to reduce mortality risk.

## INTRODUCTION

Cardiovascular diseases (CVDs), namely, heart disease and stroke, are leading causes of death in Japan.^1^ Furthermore, because stroke is a major cause of certification for long-term care insurance in Japan,^1^ risk factors for stroke also contribute to a decline in activities of daily living (ADL). Therefore, the prominent risk factors for CVD must be identified if we are to lower the risks for mortality and ADL decline. The population attributable fraction (PAF) is an estimate of the burden of a disease.^2^ My colleagues and I estimated the PAFs of all-cause death, CVD death, CVD incidence, ADL decline, and smoking-related diseases due to established CVD risk factors,^3^^–^^10^ and the results are described herein.

## METHODS

### Cohort studies

#### Ohsaki Cohort Study

The setting and design of the Ohsaki Cohort Study have been reported in detail elsewhere.^11^ In brief, this prospective cohort study started in 1994. A self-administered questionnaire requesting information on various health-related lifestyles was delivered to all National Health Insurance (NHI) beneficiaries aged 40 to 79 years living in the catchment area of the Ohsaki Public Health Center, Miyagi Prefecture, Japan. In Japan, the NHI is used by farmers, the self-employed, pensioners, and their dependents. The Ohsaki Public Health Center, which is a local government agency, provides preventive health services for the residents of 14 municipalities. The questionnaires were delivered to and collected from the subjects’ residences by public health officials in each municipality. This procedure yielded a high response rate of 94.6% (*n* = 52 029). A total of 776 subjects were excluded from the study because they had withdrawn from the NHI before 1 January 1995, when the prospective collection of NHI claim files began. Thus, 51 253 subjects formed the study cohort. Among the participants in the Ohsaki NHI Cohort Study, 16 515 (32.2%) underwent an annual health check-up between April and December 1995, and they provided their consent for the use of the results in the present study.

#### EPOCH-JAPAN

The EPOCH-JAPAN Study is a pooled analysis of 13 cohort studies that are examining the relation between health measures (laboratory measures plus lifestyle and behavioral factors) and disease (mortality and incidence) in the Japanese population. To be included in the meta-analysis, a study had to collect data on health examination measures, have a follow-up of at least 10 years, and enroll more than 1000 participants. Both nationwide and single-site cohort studies were included. Inclusion criteria for participants were age at entry (age 40–90 years) and availability of data on sex, age at entry, systolic blood pressure, and diastolic blood pressure. Because the end of follow-up varied between cohorts, age range during follow-up was limited to between 40 and 90 years, and the end of the observation period was set at age 90 years.

#### NIPPON DATA80

The subjects of this cohort study participated in the National Cardiovascular Survey of 1980. The standardized procedures used in that survey have been described elsewhere.^12^ All household members aged 30 years or older were surveyed in 300 randomly selected census tracts throughout Japan.

The number of individuals selected was 13 771. Among these, 10 546 had complete baseline information on age, sex, and blood pressure (BP). The sample comprised the National Integrated Project for Prospective Observation of Noncommunicable Disease and Its Trends in the Aged (NIPPON DATA80).

#### Miyagi Cohort Study

From June through August 1990, self-administered questionnaires on health habits were delivered to 51 291 subjects who were aged 40 to 64 years and lived in 14 municipalities of Miyagi Prefecture, in northern Japan. Usable questionnaires were returned by 47 605 subjects, yielding a response rate of 91.7%.

#### CARDIA Study

The CARDIA study, a biethnic, prospective, multicenter epidemiologic study of the evolution of risk factors in young adults, has been described in detail elsewhere.^13^ Briefly, from 1985 to 1986, 5115 African-American and white adults aged 18 to 30 years were examined in Birmingham, AL, Chicago, IL, Minneapolis, MN, and Oakland, CA. At the Birmingham, Minneapolis, and Chicago sites, participants were randomly selected from total communities or from specific census tracts. In Oakland, participants were randomly selected from members of the Kaiser Permanente Medical Care Program. At each site, recruitment achieved nearly equal numbers with respect to race (African American, white), sex, education (high school or less, more than high school), and age (18–24 years, 25–30 years). Fifty percent of invited individuals contacted were examined (47% of African Americans and 60% of whites) and formed the CARDIA cohort.

#### ARIC Study

The ARIC Study is a multicenter prospective cohort study investigating the natural history of atherosclerotic disease in the US communities of Forsyth County, NC, Jackson, MI, Washington County, MD, and the northwest suburbs of Minneapolis, MN.^14^ At baseline, in 1987–89, the cohort comprised 15 792 men and women aged 45 to 64 years who were selected by using a list or area probability sampling. Race/ethnicity was self-reported; only African Americans were recruited in the Jackson study center. The baseline home interview assessed participant sociodemographic characteristics, smoking and alcohol-drinking habits, medication use, and personal history of diseases.

### Calculation of population attributable fraction

PAF was calculated using the formula^2^: PAF = pd × (relative risk − 1)/relative risk, where pd is the proportion of cases exposed to the risk factor.

## RESULTS

### Relationship of blood pressure with all-cause mortality and CVD mortality^3^

To determine the impact of high BP on CVD mortality and all-cause mortality, We investigated the relationships of BP category with CVD mortality and estimated PAFs using data from the Ohsaki Cohort Study. In accordance with the Joint National Committees Seventh Report (JNC7),^15^ hypertension (HT) was defined as a systolic BP of 140 mm Hg or higher, a diastolic BP of 90 mm Hg or higher, or current use of antihypertensive medication. Participants who did not satisfy the HT criteria but had a systolic BP of 120 mm Hg or higher or a diastolic BP of 80 mm Hg or higher were regarded as having prehypertension (pre-HT). Those who satisfied neither set of criteria were regarded as having normal BP. A multivariate-adjusted Cox proportional hazards model was used to estimate the hazard ratio (HR) of CVD mortality associated with BP status. During 12 years of follow-up, 321 participants died of CVD. Because the positive relationship between BP and CVD mortality was steeper in middle-aged (age 40–64 years) adults than in elderly (age 65–79 years) adults, the PAF of CVD mortality was calculated separately for these groups. The HRs (95% confidence interval [CI]) for CVD mortality for pre-HT and HT were 1.31 (0.59–2.94) and 2.98 (1.39–6.41), respectively, for middle-aged adults and 1.03 (0.62–1.70) and 1.65 (1.02–2.64) for elderly adults. Adults with either pre-HT or HT accounted for 47% and 26% of CVD deaths among middle-aged and elderly participants, respectively. Similarly, nonoptimal BP explained 18.9% and 4.6% of all-cause deaths among middle-aged and elderly participants, respectively.

### Relationship between BP and all-cause mortality^4^

To determine sex- and age-specific HRs and the effect of BP on all-cause mortality, and to estimate the contribution of high BP to all-cause death, a meta-analysis of data from 13 population-based cohort studies in Japan was conducted (EPOCH-JAPAN). Poisson regression was used to estimate all-cause mortality rates and ratios. In the model, BP data were treated as continuous (increments of 10 mm Hg) and categorical (every 10 mm Hg), in accordance with the JNC7 recommendations.^15^ Potential confounders included body mass index (BMI), smoking, alcohol consumption, and cohort. The impact of HT was measured using PAFs. The adjusted mortality rate rose as BP increased, and the trend was more distinct in younger men and women. The trend in HRs was similar and more apparent in younger men (HR for an increase in BP of 10 mm Hg in men aged 40–49 years: systolic BP 1.37, 95% CI 1.15–1.62; diastolic BP 1.46, 95% CI 1.05–2.03) than in older men (age 80–89 years: systolic BP 1.09, 95% CI 1.05–1.13; diastolic BP 1.12, 95% CI 1.03–1.22). The PAF of HT was 22.7% in men and 17.9% in women when normal BP was defined as the reference level and 11.9% in men and 10.9% in women when the pre-HT group was defined as the reference level.

### Relationship between BP and subsequent decline in ADL^5^

To determine the relationship between baseline BP in 1980 and ADL in 1999 among a general population of Japanese aged 47 to 59 years, We analyzed the NIPPON DATA80 dataset. Using 1999 ADL data, We compared data from NIPPON DATA80 survivors without (*n* = 1816) and with (*n* = 75) impaired ADL, using baseline BP information collected in 1980. Multiple-adjusted logistic regression analyses were used to estimate the risk of impaired ADL according to baseline BP category, as described in the JNC7 guidelines.^15^ Stage 2 HT was defined as a systolic BP of 160 mm Hg or higher, a diastolic BP of 100 mm Hg or higher, or use of antihypertensive medication. Participants who did not satisfy the stage 2 HT criteria but had a systolic BP of 140 mm Hg or higher or a diastolic BP of 90 mm Hg or higher were regarded as having stage 1 HT. Those who did not satisfy HT criteria but had a systolic BP of 120 mm Hg or higher or a diastolic BP of 80 mm Hg or higher were regarded as having pre-HT. Those who satisfied none of the above sets of criteria were regarded as having normal BP. Excess impaired ADL due to nonoptimal BP was calculated. As compared with the normal BP category, the adjusted odds ratio (OR) of having impaired ADL was higher among those with pre-HT (OR, 1.50; 95% CI, 0.55–4.09), stage 1 HT (OR, 1.56; 95% CI, 0.56–4.32), and stage 2 HT (OR, 2.96; 95% CI, 1.09–8.05). Nonoptimal BP explained 45% (33.7/75) of impaired ADL. Blood pressure categories with a composite of mortality were also positively associated with impaired ADL.

### Combined effect of hypertension and cigarette smoking on all-cause mortality and CVD mortality^6^

To describe the fraction of CVD mortality and all-cause mortality that could be explained by current tobacco consumption and HT in Japan, we calculated the age-specific combined effect of smoking and HT on CVD mortality and all-cause mortality in NIPPON DATA80, which followed a representative cohort of 8912 Japanese men and women without a history of stroke or heart disease. Participants were categorized as a nonsmoker without HT, current smoker only, HT only, or current smoker with HT. Hypertension was defined as a systolic BP of 140 mm Hg or higher, a diastolic BP of 90 mm Hg or higher, or current use of antihypertensive medication.^15^ The PAFs of CVD mortality and all-cause mortality were calculated based on relative hazards assessed using proportional hazards regression models. During 19 years of follow-up, there were 313 and 291 CVD deaths and 948 and 766 all-cause deaths among men and women, respectively. The PAFs of CVD mortality due to smoking or HT were 35.1% for men and 22.1% for women. The PAF of CVD mortality was higher in participants younger than 60 years (57.4% for men and 40.7% for women) than in those who were older (26.3% for men and 18.1% for women).

### Relationship between cigarette smoking and all-cause mortality^7^

To examine the relationship between smoking and all-cause mortality and estimate the PAF for all-cause death due to cigarette smoking, 18 945 men and 17 107 women (age 40–64 years) in the Miyagi Cohort study were followed. The relative risk (RR) of mortality was estimated using Cox regression according to smoking category, with adjustment for age, education, marital status, history of diseases, alcohol consumption, BMI, walking, and dietary variables. A total of 1209 men and 499 women died during the 11-year follow-up period. Multivariate RRs of all-cause mortality for current smokers as compared with those of never smokers were 1.71 (95% CI, 1.44–2.03) for men and 1.44 (95% CI, 1.06–1.94) for women. Of all deaths, 34% among men and 4% among women were attributable to current or past smoking.

### Relationship between tobacco consumption and self-reported disease before middle age^8^

Evidence of harm from cigarette smoking during young adulthood is limited. We assessed associations between cigarette smoking and several self-reported illnesses in a prospective cohort study of healthy young adults. The data were derived from 4472 adults who participated in the CARDIA study. They were aged 18 to 30 years at baseline and were reexamined at least once after 7, 10, or 15 years. Tobacco consumption in 1985–86 was related to self-reported smoking-related cancers, circulatory disease, and peptic ulcer. The incidence of these diseases was 9.3 per 1000 person-years among current smokers and 4.5 per 1000 person-years among those who had never smoked and had no exposure to passive smoke; the relative risk (adjusted for race, sex, education, and center) was 1.96 (95% CI, 1.42–2.70). Assuming a causal relationship, 32% of these premature incidents were attributable to smoking. The relative risks of liver disease, migraine headache, depression, being ill the day before the examination, chronic cough, and phlegm production were also higher among smokers.

### Low-risk profiles for cardiovascular disease incidence and mortality in a US population^9^

A large proportion of CVD events among white Americans can be explained by borderline or elevated levels of CVD risk factors. The degree to which this is true among African Americans is unclear. Thus, to determine the proportion of such events, We analyzed data from the ARIC Study, which included 14 162 middle-aged adults who were free of recognized stroke or coronary heart disease and had baseline information on risk factors. Based on national guidelines, risk factors (BP, cholesterol levels, diabetes, and smoking) were categorized as optimal, borderline, or elevated.^15^^–^^17^ The incidences of CVD (a composite of stroke and coronary heart disease; *n* = 1492), CVD mortality (*n* = 612), and all-cause mortality (*n* = 1824) were determined for a 13-year period. Overall, 6.2% and 70.2% of CVD incidence was explained by borderline and elevated risk, respectively. Similarly, 5.3% and 81.5% of CVD deaths were explained by borderline and elevated risk, and 7.2% and 64.5% of all-cause deaths were explained by borderline and elevated risk.

### Low-risk profile for all-cause mortality and cardiovascular disease in a Japanese population^10^

Studies have focused on low-risk profiles for CVD in Europe and North America,^9^ but few have examined the long-term low-risk profile for CVD among the Japanese general population. The present study examined whether having a favorable risk factor profile yields lower all-cause mortality and whether the proportion of adults with a low-risk profile is larger among the Japanese population. The data were derived from NIPPON DATA80. A total of 8339 men and women who were aged 30 to 69 years and had no history of cardiovascular diseases were followed for 19 years. Low risk was defined as having all of the following baseline characteristics: a systolic BP lower than 120 mm Hg, a diastolic BP lower than 80 mm Hg, no antihypertensive medication, serum cholesterol 160 to 240 mg/dL (4.14–6.22 mmol/L), no history of diabetes, and no tobacco consumption. The long-term mortality of the low-risk group was compared with that of other groups using a Cox proportional hazards model. Overall, 9.4% of participants were classified as low risk. The multivariate-adjusted HR among low-risk individuals as compared with others was 0.33 (95% CI, 0.15–0.74) for CVD and 0.63 (95% CI, 0.46–0.88) for all-cause mortality. The PAF associated with the elevated risk profile was 66% for CVD mortality and 36% for all-cause mortality. The greatest attributable risk factor for all-cause mortality was high BP. In conclusion, rates of all-cause and CVD mortality were lower among Japanese individuals with a favorable cardiovascular disease risk profile.

## DISCUSSION

The PAFs of established risk factors were estimated for several endpoints (Table). These risk factors significantly contributed to the outcomes, especially high BP and smoking.

**Table.** Summary of Population Attributable Risk Fractions (PAFs) of cardiovascular disease (CVD) mortality, all-cause mortality, and other endpoints

Risk factor	Definition of elevated risk	Subgroup	PAF of CVD mortality	PAF of all-cause mortality	PAF of other endpoints	Setting	Reference no.
Abnormal BP	SBP ≥120 mm Hg, DBP ≥80 mm Hg, or antihypertensive medication	40–64 y	47%	19%		Ohsaki Cohort Study	^3^
		65–79 y	26%	5%			

	SBP ≥120 mm Hg, DBP ≥80 mm Hg, or antihypertensive medication	Men		23%		EPOCH-JAPAN	^4^
		Women		18%			

	SBP ≥120 mm Hg, DBP ≥80 mm Hg, or antihypertensive medication				Impaired ADL, 45%	NIPPON DATA80	^5^

Combined effect of hypertension and smoking	Hypertension: SBP ≥140 mm Hg, DBP ≥90 mm Hg, or antihypertensive medication. Smoking: current smoking	40–59 y men	57%	29%		NIPPON DATA80	^6^
		40–59 y women	41%	7%			
		60 y– men	26%	25%			
		60 y– women	18%	18%			

Cigarette smoking	Ever smoker (Current + past)	Men		34%		Miyagi Cohort Study	^7^
		Women		4%			

	Ever smoker + passive smoker				Incidence of self-reported smoking-related diseases,^a^ 32%	CARDIA Study	^8^

Any nonoptimal risk factor	Nonoptimal BP: SBP ≥120 mm Hg, DBP ≥80 mm Hg, or antihypertensive medication	Overall	87%	72%	CVD incidence, 76%	ARIC study	^9^
	Total cholesterol ≥200 mg/dL						
	Diabetes: Fasting serum glucose ≥126 mg/dL, random serum glucose ≥200 mg/dL, or physician diagnosis of or treatment for diabetes						
	Smoking: Ever smoker						

	Nonoptimal BP: SBP ≥120 mm Hg, DBP ≥80 mm Hg, or antihypertensive medication	Overall	66%^b^	33%^b^		NIPPON DATA80	^10^
	Total cholesterol <160 mg/dL or ≥240 mg/dL						
	Diabetes: Fasting serum glucose ≥126 mg/dL, random serum glucose ≥200 mg/dL, or physician diagnosis of or treatment for diabetes						
	Smoking: Current smoking						

BP: blood pressure; SBP: systolic BP, DBP: diastolic BP, ADL: activities of daily living.

^a^Smoking-related diseases: Smoking-related cancer, cardiovascular disease, and peptic ulcer.

^b^Calculated from original article (Ref. 10).

In the Ohsaki study, 47% and 26% of CVD deaths among middle-aged and elderly participants, respectively, were explained by nonoptimal BP. Similarly, approximately 20% of all-cause mortality was explained by nonoptimal BP in the EPOCH-JAPAN Study. These findings are similar to those of other Japanese studies. Sairenchi et al reported that in middle-aged adults, 60% (men) and 15% (women) of CVD deaths were explained by nonoptimal BP; the corresponding values in elderly adults were 28% and 7%.^18^ Ikeda et al reported that 38% of total CVD mortality in men and 36% in women would be prevented by elimination of high-normal to severe hypertension.^19^ Thus, better management of high BP is necessary because the PAFs for all-cause and CVD mortality due to hypertension were high, especially in younger adults. However, there are several problems regarding the management of high BP. Although antihypertensive medications can potentially prevent CVD,^15^ the rate of BP control has been insufficient.^15^^,^^20^ One report showed that only 24% of adults with untreated hypertension at routine health check-ups had started treatment within the subsequent year.^21^ Thus, more effort should be directed toward primary prevention, early detection, early treatment, and better control of high BP.

The PAF for all-cause mortality due to tobacco consumption is high in Japan,^5^ especially among men. Hara et al reported that 22.2% of all-cause mortality was explained by smoking,^22^ and Uno et al reported that 24.9% of all-cause deaths were explained by smoking.^23^ Although the smoking rate is generally decreasing in Japan,^1^ it is still higher than in other developed countries. Efforts to decrease smoking rates should continue.

We found that having any one nonoptimal risk factor explained large proportions of all-cause and CVD deaths.^9^^,^^10^ In Japan, the combined effect of smoking and hypertension was extremely large,^6^ which highlights the importance of combating smoking and hypertension. A recent report showed that the PAFs for all-cause and CVD deaths due to high BP and smoking were higher than those due to diabetes and suboptimal serum cholesterol.^10^ However, the baseline survey was performed 30 years previously (1980); surveys conducted more recently show that the prevalence of both obesity and diabetes has increased among Japanese men.^1^ Further studies that estimate PAFs using more recent baseline data are needed in order to assess the burden of these diseases.

In previous studies, my colleagues and I mainly used Cox proportional hazards models to estimate hazard ratios and inserted them into the traditional formula to calculate PAFs, as was done in several cohort studies.^24^^,^^25^ However, recent publications have refined the calculation of PAFs derived from the Cox model^26^^,^^27^ when a such model is applied to a cohort study. Further discussion and refinement will be necessary to establish PAF estimation in the cohort study setting.

In conclusion, CVD risk factors, especially BP and cigarette smoking, explain most all-cause and CVD mortality. Prevention, early detection, and treatment of these conventional risk factors are required to reduce the risk for mortality.
